# Study on load transfer mechanisms and impact on adjacent railway subgrade within loess regions

**DOI:** 10.1016/j.heliyon.2024.e30085

**Published:** 2024-04-26

**Authors:** Wenhui Zhao, Ke Zhang, Jinjiang Xu, Xueli Liu

**Affiliations:** School of Civil Engineering, Lanzhou Jiaotong University, Lanzhou, 730070, China

## Abstract

Clarifying load transfer mechanism and the influence of the widening of a newly-built railway is the premise for the construction of adjacent project in loess region. This paper uses monitoring datas obtained from three sections in different stages to analyze the distribution laws of load exert on piles and soil between piles, investigates the variation laws of filling height on the earth pressure at different excavation steps and elucidates the influence of the filling height of newly built subgrade on the existing subgrade. In addition, a fitting formula y = a(1-e^-bx^) + cx is proposed to describe the relationship between the ratio of the additional displacement to the filling height, which is applicable for similar projects.

## 1 Introduction

As China's railway network is being improved, new railways are connected with existing railways, resulting in adjacent subgrade sections. As the operation speed increases, the train becomes more sensitive to changes in the Track Quality Index (TQI) of the upper track, increasing the requirements of the subgrade structure [1]. Construction projects adjacent to existing railways must ensure the comfort and safety of the train operation and control the deformation of the subgrade structure. The existing subgrade structure has settled after construction, especially in loess areas [2]. The load transfer mechanism from the newly built subgrade to the existing subgrade has to be investigated to ensure that the post-construction settlement remains within the standard and to control the influence of the newly built subgrade.

The junction between the new and existing subgrade is the most critical aspect of the adjacent subgrade project. Uneven settlement will occur if it is not constructed appropriately. The new subgrade may slide along the joint surface, causing overall subgrade instability. However, the uneven settlement is non-uniform in the vertical direction and continuous near the settlement center. Moreover, an inadequate joint may result in precipitation infiltration along cracks and the deformation and settlement of the subgrade. Step excavation and the use of a geogrid at the junction between the new and existing subgrade are commonly used, which have been investigated by several scholars. Lazorenko [3] used the finite element method to compare and analyze the reinforcement performance of a railway widening project using geosynthetics, soil nailing, and pile construction. The author examined the soil deformation during the reinforcement process, studied the failure mechanism of the slope during embankment widening, and determined the reasonable application range of reinforcement structure. Zhao et al. [4] investigated the embankment widening of an expressway using prestressed concrete piles and a geogrid at the joint between the new and existing subgrade. This method proved the ration and reduced the construction time. Motoyuki et al. [5] carried out field tests to study the feasibility of a multi-layer geotextile and clay cushion for widening a subgrade. This strategy increased the integrity of the new and existing subgrade.

The construction of new railway lines affects existing lines. After the construction of the new line subgrade, changes occur in the vertical and horizontal directions at the joint between the new and existing subgrade. Yu [6], Han [7], Liang [8], and Wang [9] used numerical methods to assess the additional stress caused by different foundation and embankment techniques (geosynthetics, lightweight embankment, deep mixed columns, and a separation wall). The settlement characteristics of the subgrade after the highway widening project were discussed, and suggestions were proposed for the type of composite foundation to reduce pavement damage. Ren [10] carried out multiple large-scale model tests to analyze the influence of the wall deformation, earth pressure distribution, reinforcement strain, and potential fracture surface caused by different connection types and relative densities when a shored mechanically stabilized earth (SMSE) wall was used to widen a subgrade. Miao [11] used a physical model test to analyze the influence of geogrid reinforcement on the differential settlement of the embankment in a highway widening project. The result showed that the geogrid layer stabilized the soil arch in the embankment, reduced the differential settlement of the embankment surface, and improved the performance of the widened embankment. Lu [12] conducted field tests to monitor the earth pressure at different positions. They quantified the soil arching effect using the stress concentration ratio and assessed the soil arching ratio and the geomembrane performance to determine the load redistribution mechanism caused by the soil arch.

Loess is a unique soil type prone to deformation due to stress or high moisture [13]. Few studies have investigated railway widening projects in loess areas. Thus, it is unclear which structures provide the optimum performance. In this study, step excavation is performed, and a geogrid is used on the slope of an existing subgrade according to the TB 10001-2016 Code for the Design of Railway Earth Structures to provide a suitable joint between the new and existing subgrade. Three representative sections are selected for analysis. Monitoring data in different stages are used to assess the load distribution of the piles and the soil between the piles under the subgrade load and the mechanism of pile-soil load sharing. The effect of the filling height on the earth pressure at different stages of the step excavation is evaluated. The amplitude of earth pressure at different stages of step is clarified. The influence of the newly built subgrade on the existing subgrade is examined, and curve fitting is performed to determine the effect of the filling height on the displacement and deformation of the existing subgrade to assess the structure's performance.

## 2 Overview of the adjacent subgrade project

### 2.1 Engineering geological conditions

The lithology of the stratum at this site is predominantly sandy loess, gravelly sand, and underlying mudstone with sandstone. The physical and mechanical parameters of the strata are listed in Table 1. The engineering geological characteristics are as follows:

②_422_Sandy loess ($Q_{4}^{al+pl3}$): light yellow, wet, slightly dense, uniform soil; visible pinhole macropores and wormholes; thickness of 2.0–3.5 m. Grade II ordinary soil. $\sigma_{0}=120kPa$. $E_{s0.1-0.2}=4.2MPa$.
②_423_ Sandy loess ($Q_{4}^{al+pl3}$): light yellow, saturated, slightly dense; layer thickness 7.3–12.4 m. Grade II ordinary soil. $\sigma_{0}=80kPa$. $E_{s0.1-0.2}=3.0MPa$, $E_{s0.2-0.3}=3.7MPa$.
②_933_Gravel sand ($Q_{4}^{al+pl5}$): light yellow, saturated, slightly dense to medium dense; mainly composed of quartz and feldspar; thickness of 0–7.2 m. Grade I loose soil. $\sigma_{0}=250kPa$.
②_1033_Fine round gravel soil ($Q_{4}^{al+pl6}$): variegated, saturated, medium density; mainly composed of sandstone; particle size: 2–20 mm (50 %), greater than 20–60 mm (10 %); thickness of 0–2.8 m. Grade II ordinary soil. $\sigma_{0}=400kPa$.
⑨42Mudstone with sandstone ($N_{1}^{Ms+Ss}$): brownish red and strongly weathered; joint fissures. Grade IV soft stone. $\sigma_{0}$ = 300kPa.

**Table 1:** Physical and mechanical parameters of strata.

Level number	Stratum name	γ/(kN·m^−3^)	Poisson ratio	Cohesion/kPa	Internal friction angle/(°)
②_422_	Sandy loess	18.5	0.32	15.2	21.1
②_423_	Sandy loess	18.7	0.32	18.4	21.4
②_933_	Gravelly sand	19.3	0.31	5.2	23.5
②_1033_	Fine round gravel soil	19.5	0.31	8.1	24.5
⑨_42_	Mudstone with sandstone	19.8	0.33	13.1	23.1

### 2.2 Working conditions in the adjacent subgrade section and monitoring section

**Fig. 1:**
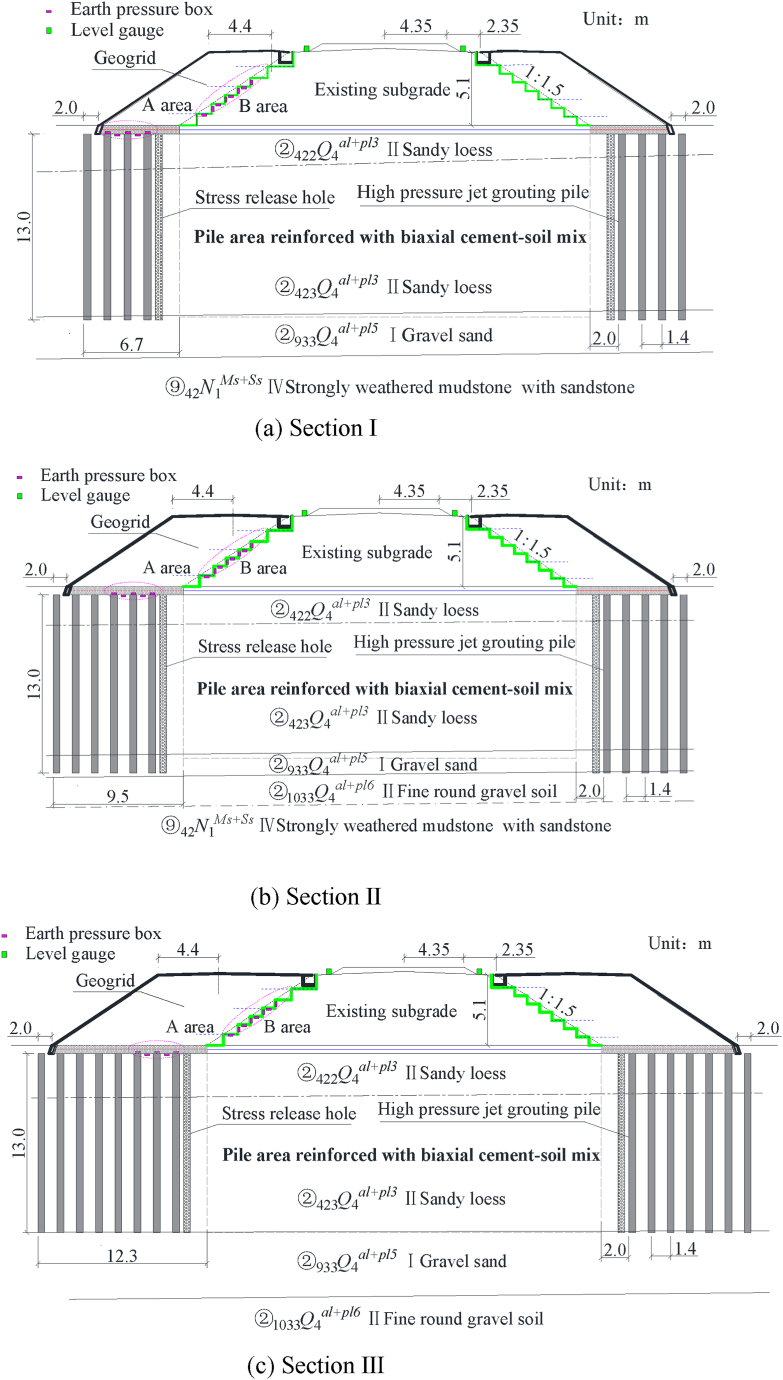
Three typical sections.

The study area is located in Shuping Town, Yongdeng County, Lanzhou City. The three typical sections analyzed in this study are shown in Fig. 1. The composite foundation with the piles is reinforced with a biaxial cement-soil mix. The pile diameter is 50 cm, the pile length is 13 m, and the pile spacing is 1.3 m. The design bearing capacity of the composite foundation is 150 kPa. The pile top is covered by a 0.5 m thick open-graded crushed stone cushion, and a biaxial geogrid layer is located in the middle of the cushion. The layers of the subgrade from top to bottom include graded crushed stone (0.6 m), a mixture of soil and 4 % cement (1.9 m), and seepage soil. The biaxial geogrid with a width of more than 3 m is placed on the excavation steps.

A composite foundation manufactured by high-pressure jet grouting pile is used in the new subgrade. The pile diameter is 50 cm, the pile length is 13 m, and the pile spacing is 1.4 m. The piles are square arrangement, and the bearing capacity of the composite foundation is 155 kPa. A stress release hole is located 1.45 m from the toe of the slope. The pile diameter is 0.5 m, and the longitudinal spacing is 1.2 m. The hole is filled with crushed stone and covered with 3:7 lime soil after mechanical excavation. The surface layer of the subgrade, the bottom layer of the subgrade, and the area below the immersion protection consist of 0.6 m graded crushed stone +0.1 m medium-coarse sand + a composite geomembrane +0.1 m medium-coarse sand, an anti-frost heave water seepage filler (0.8 m, permeability coefficient >5 × 10^−5^ m/s), and seepage soil (the same as the bottom layer of the subgrade), respectively.

**Fig. 2:**
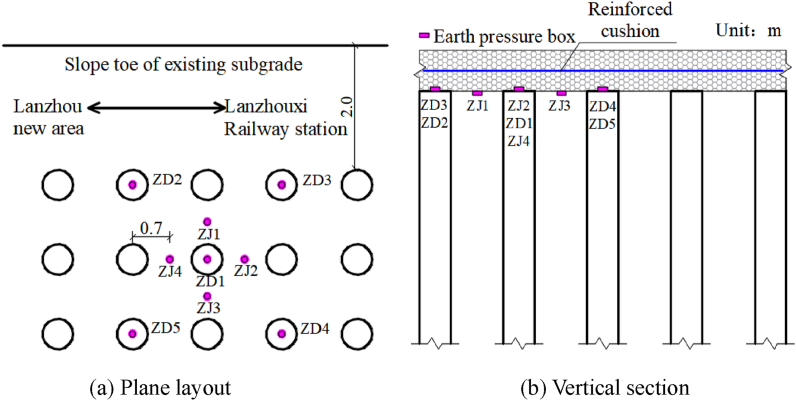
The location of the earth pressure box at the top of the pile.

**Fig. 3:**
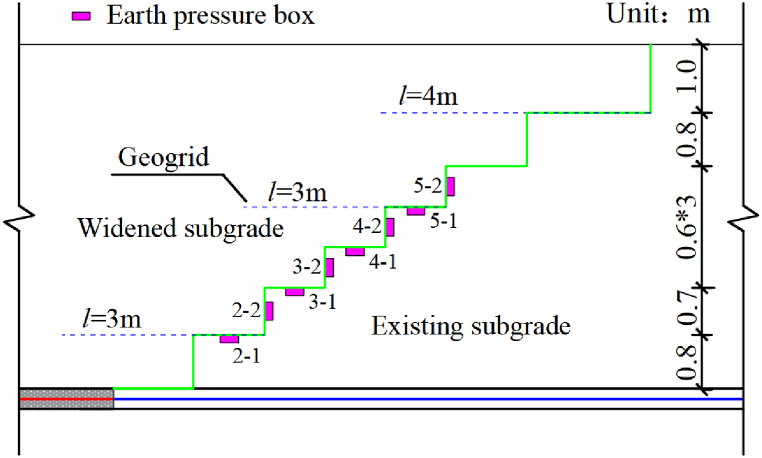
The layout of the earth pressure boxes at the steps.

As shown in Fig. 1, a level gauge is placed longitudinally at the toe of the ballast slope of the existing subgrade before the construction of the new subgrade. It is used to evaluate the influence of constructing the new subgrade on the existing subgrade. As shown in Fig. 2, earth pressure boxes are located at the pile top and between the piles of the new composite foundation adjacent to the existing subgrade. Fig. 3 shows the earth pressure boxes located at the steps.

### 2.3 Moisture content and static load tests of the single-pile composite foundation

Several sections are selected to measure the moisture content in the widened section. The moisture contents of the strata are listed in Table 2.

**Table 2:** The moisture contents of the strata.

Stratum name	Moisture content
②_422_Sandy loess	15.6–33.6
②_423_Sandy loess	23.5–37.3
②_933_Gravel sand	25.6–36.7
②_1033_Fine round gravel soil	25.1–36.5

**Fig. 4:**
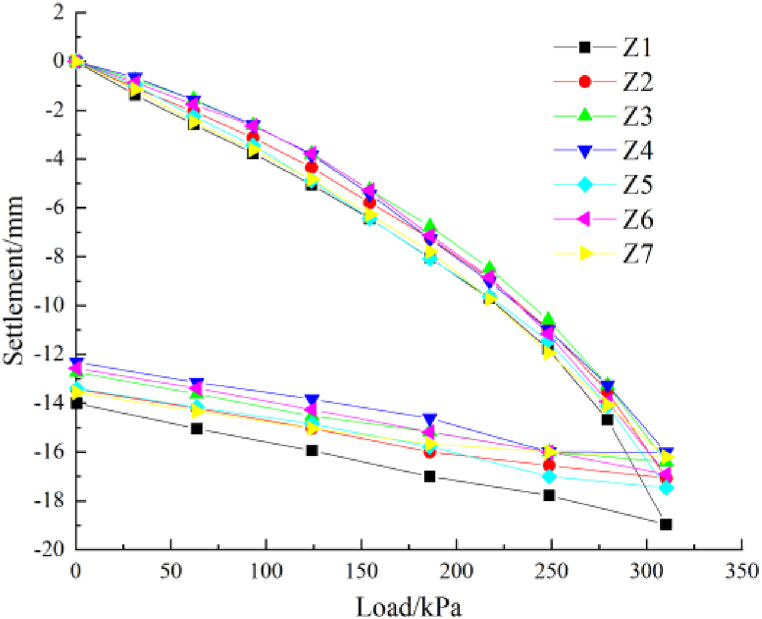
The load-settlement curves for the widened section.

Seven high-pressure jet grouting piles were selected for the load test of the single-pile composite foundation to determine whether the bearing performance met the design requirements after 28 days. The maximum load in the field test was 310 kPa. The slow maintenance loading method was used to apply the load in ten stages. After the application of each load, unloading occurred in five stages, and the total rebound was recorded. The load-settlement curve is shown in Fig. 4, and the results are listed in Table 3. The maximum bearing capacity is 209.17 kPa, meeting the design requirements (155 kPa).

**Table 3:** Results of the single-pile composite foundation bearing test.

Location	Maximum settlement/mm	Maximum rebound amount/mm	Maximum rebound rate/mm	Maximum bearing capacity/kPa
Z1	18.91	4.90	25.91	192.16
Z2	17.06	3.69	21.63	208.30
Z3	16.41	3.67	22.36	215.22
Z4	16.02	3.72	23.22	206.13
Z5	17.40	4.03	23.16	193.00
Z6	16.89	4.90	25.91	209.71
Z7	16.15	2.55	15.79	195.86

## 3 Results and discussion

### 3.1 The load distribution on top and between the piles under the embankment load

The cushion at the top of the composite foundation adjusts the proportion of pile-soil load sharing and transfers the soil load between the piles to the pile top, improving the piles' and composite foundation's bearing capacities and reducing the settlement.

**Fig. 5:**
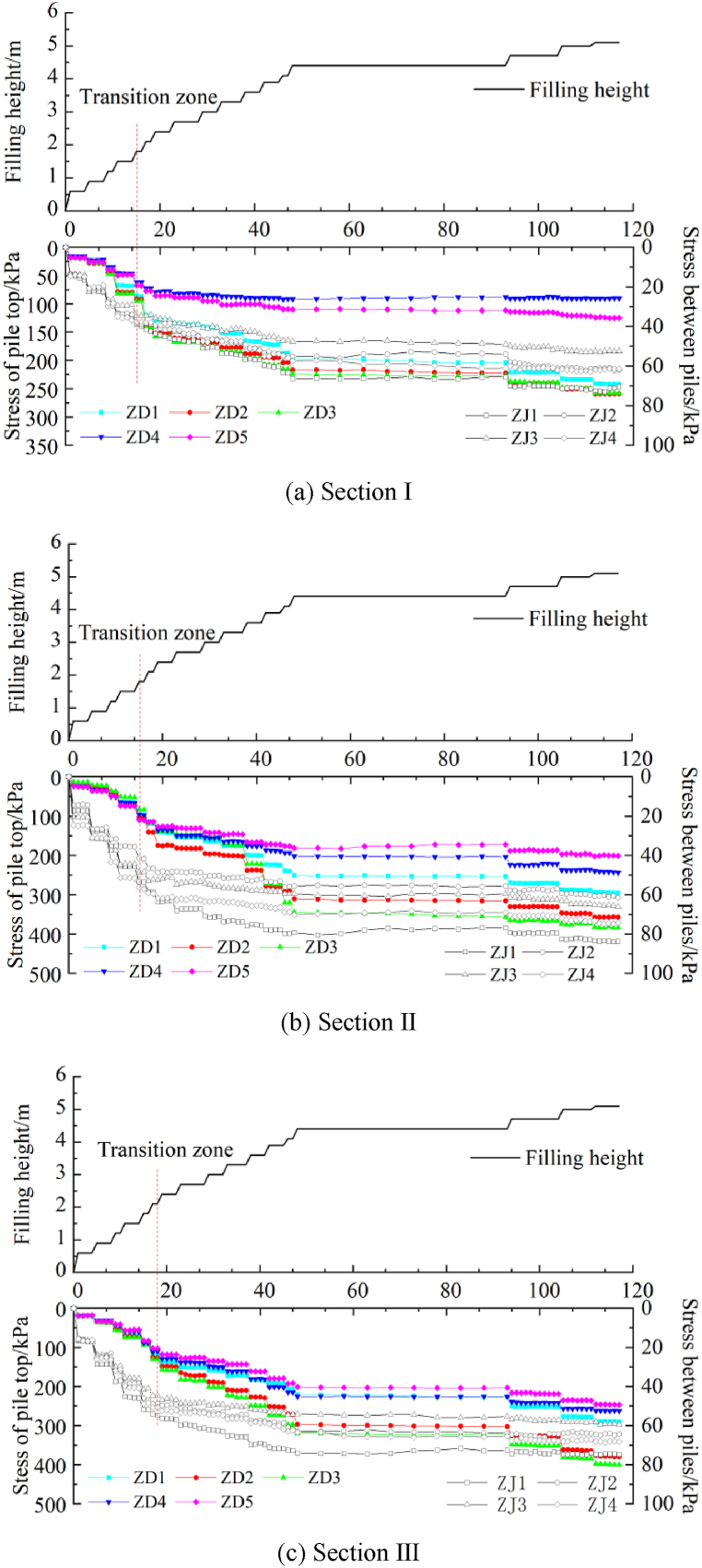
Relationship between filling height and stress at the pile top and between the piles in three sections.

**Fig. 6:**
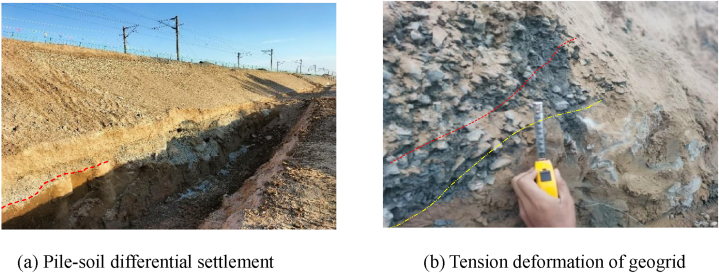
Different settlements of the pile and soil and tensile deformation of the geogrid during the excavation of the slope toe.

Fig. 5 shows the relationship between the filling height and stress at the pile top and between the piles in three sections. The stress at the pile top and the soil stress between the piles in sections I to III increases with the subgrade filling height, but there are some differences in the rate of increase. When the filling is completed, the stress at the pile top in section I is 242.459 kPa, 259.148 kPa, 257.867 kPa, 90.129 kPa, and 124.654 kPa, and the soil stress between the piles is 70.345 kPa, 62.047 kPa, 52.440 kPa, and 61.457 kPa. The stress of the pile top in section II is 295.016 kPa, 357.157 kPa, 383.085 kPa, 243.781 kPa, and 200.855 kPa, and the soil stress between the piles is 83.784 kPa, 61.521 kPa, 65.997 kPa, and 74.215 kPa. The stress of the pile top in section III is 290.370 kPa, 379.985 kPa, 399.985 kPa, 262.658 kPa, and 246.983 kPa, and the soil stress between the piles is 74.818 kPa, 64.328 kPa, 59.489 kPa, and 68.165 kPa. In sections I and II, the stress is the equivalent at ZD2 and ZD3, followed by ZD1, ZD4, and ZD5, showing a decrease in the stress with the increasing distance from the existing subgrade. In section III, the stress is the same at ZD2, ZD3, and ZD1 and is higher than that at ZD4 and ZD5. As the distance from the existing subgrade increases, the stress remains the same initially and then decreases. The soil stress between the piles in section Ⅰ and Ⅲ is the largest ZJ1 and equivalent at ZJ2 and ZJ4, ZJ3 is the smallest. In section Ⅲ, the stress at ZJ1 is the largest, followed by ZJ4, and is higher than that at ZJ2 and ZJ3, showing a decreasing with the increasing distance from the existing subgrade. The stress at the pile top and the soil stress between the piles are similar at a filling height of less than 1.5 m. However, at a filling height of 1.8 m, the stress values differ at the pile top and between the piles, and the growth rate of the soil stress is lower between the piles than at the pile top. In the early stage of subgrade filling, the upper load is small, and the pile top and the soil between the piles bear the load together, but the difference is not large. The upper load is evenly distributed to the piles and the soil between piles. However, as the embankment filling height increases, the load is transferred from the soil between the piles to the pile top. Research [14] and field test data have indicated a critical height during embankment filling. When the filling height exceeds the critical height, the top stress of the high-pressure jet grouting pile increases rapidly, whereas the rate of increase in the soil stress between the piles decreases significantly. The critical height is the height when a soil arch has been formed. Due to the tensioned membrane effect of the geogrid and the ‘net effect’ of the soil arch and reinforced cushion, the soil load between the piles is transferred to the pile top, improving the composite foundation's bearing capacity. The necessary condition for the soil arch effect is different soil settlement rates at the pile top and between the piles under a top load. The high-pressure jet grouting pile has higher stiffness than the soil between the piles. When the upper load is large, the high-pressure jet grouting pile tends to move upward, whereas the cushion layer does not. The load causes compression deformation of the soil between the piles, resulting in different settlement rates of the soil at the top and between the piles and providing the necessary conditions for the formation of the soil arch. After the excavation for the skirting wall foundation, the different settlement rates of the pile and soil and the tensile deformation of the geogrid are observed (Fig. 6).

The 48 d–94 d of subgrade filling is the intermittent period. The filling height of the subgrade during this time was 4.4 m. The stress range at the pile top and for the soil between the piles was small in the intermittent period. The reason is the subgrade consolidation and the continuous change in the stress at the pile top and between the piles under the subgrade load as a balanced state is achieved. During the intermittent period, the stress of the soil between piles shows a slightly decreasing trend, and the soil stress at the top of the piles shows a slightly increasing trend, indicating that the soil arch has been formed at this height and the soil stress between the piles has been transferred to the top of the pile.

**Fig. 7:**
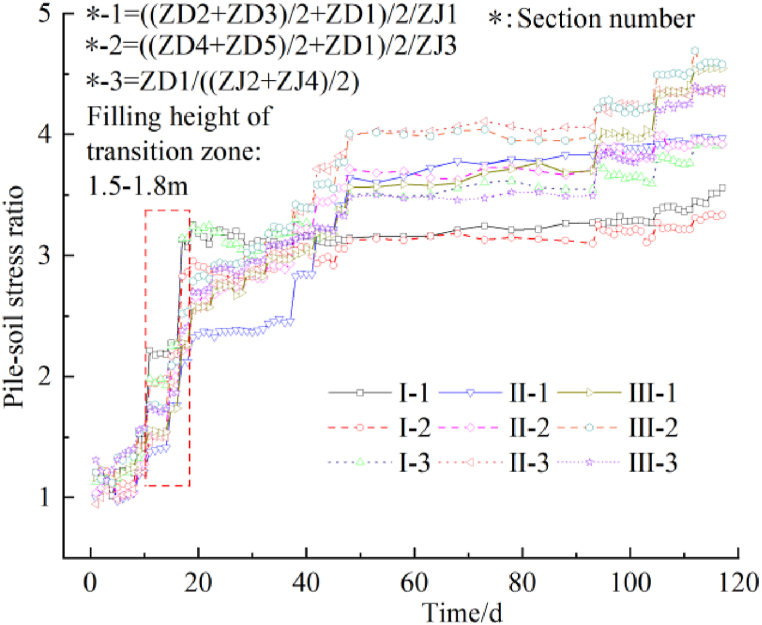
The pile-soil stress ratio over time.

Fig. 7 shows the pile-soil stress ratio over time. In the initial stage of subgrade filling, the pile-soil stress ratio in each section is close to 1.0, suggesting that the difference in the soil stress between the piles and at the top of the piles is small when the upper load is small, and the upper load is borne by the soil and the piles. As the subgrade filling height increases, a transition zone is observed. At a filling height of 1.5–1.8 m, the pile-soil stress ratio increases rapidly, indicating that the load of the soil between the piles is being transferred to the pile top, and the stress has increased at the pile top, suggesting the formation of the soil arch [15]. With the further increase in the filling height, the rate of increase in the pile-soil stress ratio in sections I to III decreases, and the changes differ in the different sections. In sections I and II, the longitudinal pile-soil stress ratio is significantly larger than the transverse one, and the pile-soil stress ratio is relatively large on the side of the existing subgrade. In section III, the longitudinal and transverse pile-soil stress ratios are equivalent, indicating that the width of the newly widened subgrade substantially influences the pile-soil stress of the lower composite foundation. When the subgrade filling is completed, the pile-soil stress ratios are 3.561, 3.336, and 3.926 in section I, 3.969, 3.919, and 4.347 in section II, and 4.547, 4.582, and 4.383 in section III, respectively. The distribution of the pile-soil ratio changes continuously when the composite foundation bears the upper load, and the pile-soil ratio is directly related to the filling height of the upper subgrade. The location of the section also affects the distribution of the pile-soil stress ratio due to the different engineering geological conditions and widths. However, the trend of the pile-soil stress ratio is similar for the different sections. As the subgrade filling height increases, a soil arch forms in the subgrade, enabling the transfer of the soil load between piles to the pile top due to the reinforced cushion.

### 3.2 The earth pressure at the steps of the new and old subgrades

The step excavation and the geogrid reinforcement connect the new and old subgrades and substantially improve the structural stability of the exisiting-widened subgrade. This method is widely used in railway widening projects. The steps transfer the additional stress generated by the widened subgrade during the filling to the existing subgrade. Horizontal and lateral earth pressure boxes were located at the steps, and stress monitoring was performed during filling to analyze the stress transfer characteristics of the steps of the new and old subgrades.

**Fig. 8:**
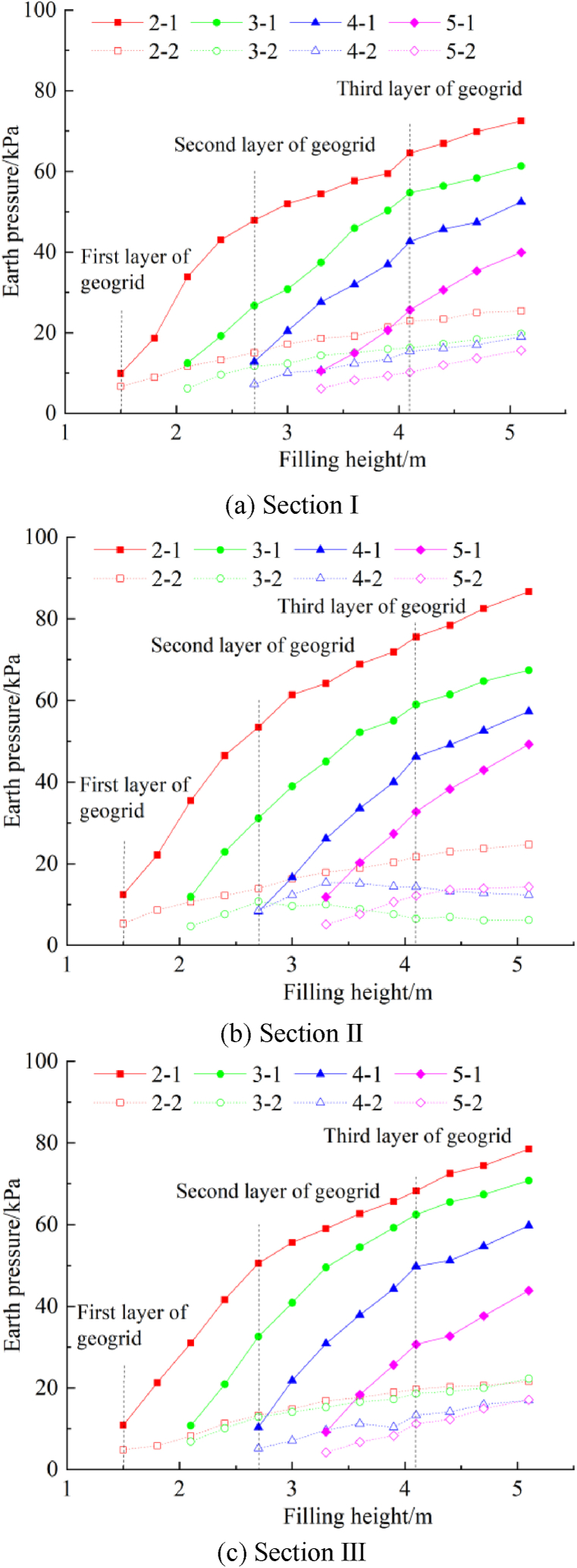
The relationship between the earth pressure at the steps and the filling height.

Fig. 8 shows the relationship between the earth pressure at the steps and the filling height. The vertical and lateral earth pressure at each step of sections I to III increases with the filling height. However, the earth pressure increases and then decreases with the filling heights at some locations. When the filling is completed, the vertical (lateral) earth pressures of the second, third, fourth, and fifth steps of section I section are 72.489 kPa, 61.348 kPa, 52.456 kPa, and 39.925 kPa (25.3787 kPa, 19.796 kPa, 18.985 kPa, and 15.637 kPa), respectively. The vertical (lateral) earth pressures of the second, third, fourth, and fifth steps of section II are 86.67 8 kPa, 67.415 kPa, 57.306 kPa, and 49.259 kPa (24.674 kPa, 6.237 kPa, 12.395 kPa, and 14.359 kPa) respectively. The vertical (lateral) earth pressures of the second, third, fourth, and fifth steps of section III are 78.532 kPa, 70.786 kPa, 59.756 kPa, and 43.851 kPa (31.633 kPa, 22.272 kPa, 16.979 kPa, and 17.127 kPa), respectively. The comparative analysis shows that the earth pressures of section II and section III are equivalent, whereas the earth pressure of section I is lower at the same position. Due to the geogrid, the growth rate of the vertical earth pressure decreases with the filling height, indicating that the geogrid disperses the stress in the upper area [16].

**Fig. 9:**
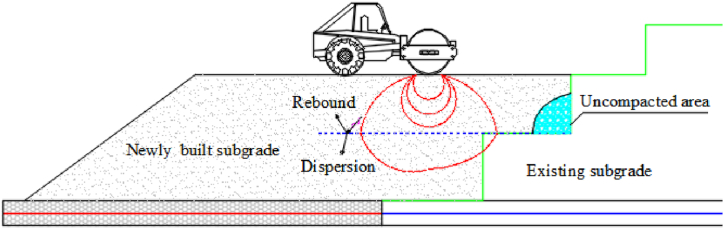
Schematic diagram of mechanical compaction.

The stress transfer at the junction of the new and old subgrade steps is directly related to the thickness of the overlying soil layer. When the overlying soil layer is thick at the steps, the vertical and lateral soil stresses are large, and vice versa. The closer the fill layer is to the vertical distance from the step, the more pronounced the effect on the step's stress. When the subgrade filling is completed, the lower step bears the load of the new and old subgrades, the vertical cohesion is higher. Because the upper step bears a smaller load, the vertical stress transfer and the cohesion are excellent, demonstrating the advantage of step excavation, which requires rolling, laying the geogrid, and other processes. During the test, the lateral earth pressure at the third and fourth steps of section II decreases with the filling height of the subgrade. Field investigations show an uncompacted area near the step (Fig. 9). When the overlying soil layer at the step reaches a certain thickness, the stress in the uncompacted area does not increase with the filling height. In contrast, the stress of the overlying soil layer decreases due to the mechanical vibration and other factors during filling. The cohesion between the new and old subgrades at the step is low, reducing the stability of the existing-widened subgrade. The plant roots and surrounding humus soil at the step should be removed, and the uncompacted area should be minimized by rolling to improve the connection between the existing and new subgrade and strengthen the structure's stability.

**Fig. 10:**
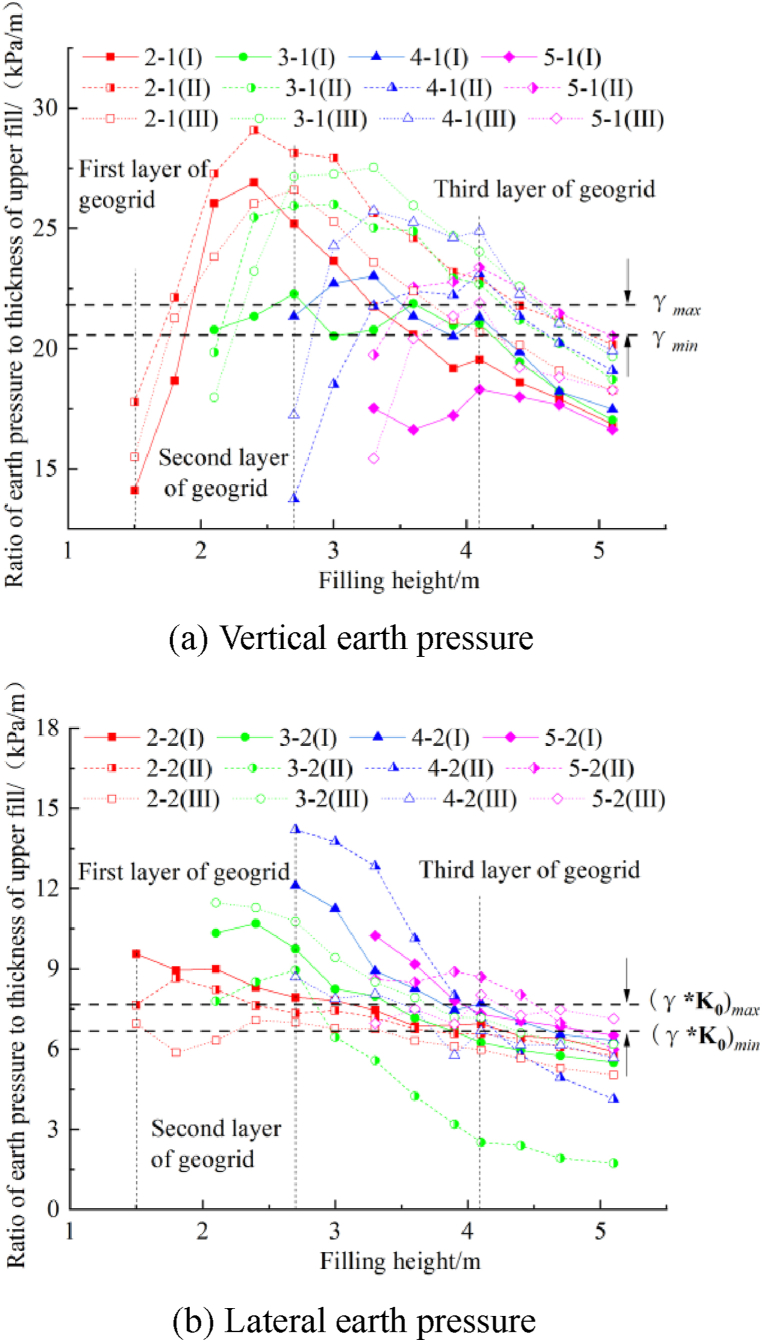
The relationship between the ratio of the earth pressure at the step to the thickness of the upper fill and the filling height.

**Fig. 11:**
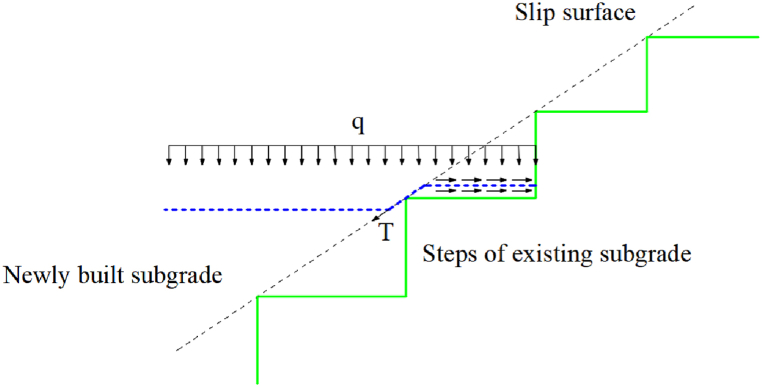
Schematic diagram of the geogrid mechanism at the junction of the existing and new subgrades.

The relationship between the ratio of the earth pressure at the step to the thickness of the upper fill and the filling height is shown in Fig. 10. The ratio of vertical earth pressure to the thickness of the upper fill increases and decreases with the filling height. The ratio decreases with an increase in the thickness of the upper geogrid. When the filling is completed, the ratio of the vertical earth pressure to the thickness of the upper fill is less than γ_min_, indicating that the geogrid reduces the vertical earth pressure at the step [17]. The ratio of the lateral earth pressure to the thickness of the upper fill is greater than (γ*K_0_)_max_ in the initial filling stage and then decreases with the filling height. When the filling is completed, only the earth pressure at the fifth step in section III is between (γ*K_0_)_min_ and (γ*K_0_)_max_. The other earth pressure values are lower than (γ*K_0_)_min_. The compactness of granular materials with a certain thickness increases when compacted by construction machinery [18,19]. Therefore, the lateral earth pressure of the upper fill increases. The compactness of filling material with a certain thickness decreases when exposed to the natural environment; thus, the upper lateral earth pressure decreases, while the lower lateral earth pressure does not change significantly. However, the geogrid reduces the horizontal stress at the junction of the subgrades, decreasing the lateral earth pressure of the step (Fig. 11).

### 3.3 Deformation of existing subgrade

In a subgrade widening project, the operation of construction machinery, subgrade filling and track loads cause stress and deformation to the existing subgrade, affecting the smoothness of the existing track and threatening the safe operation of trains. The deformation of the existing subgrade was monitored in real time to evaluate the influence of construction on the deformation of the existing line.

**Fig. 12:**
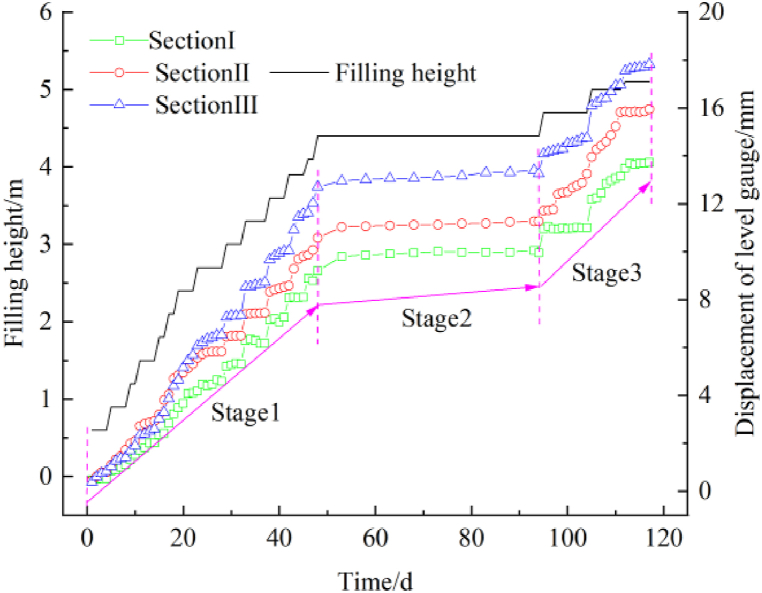
The displacement and filling height over time.

**Fig. 13:**
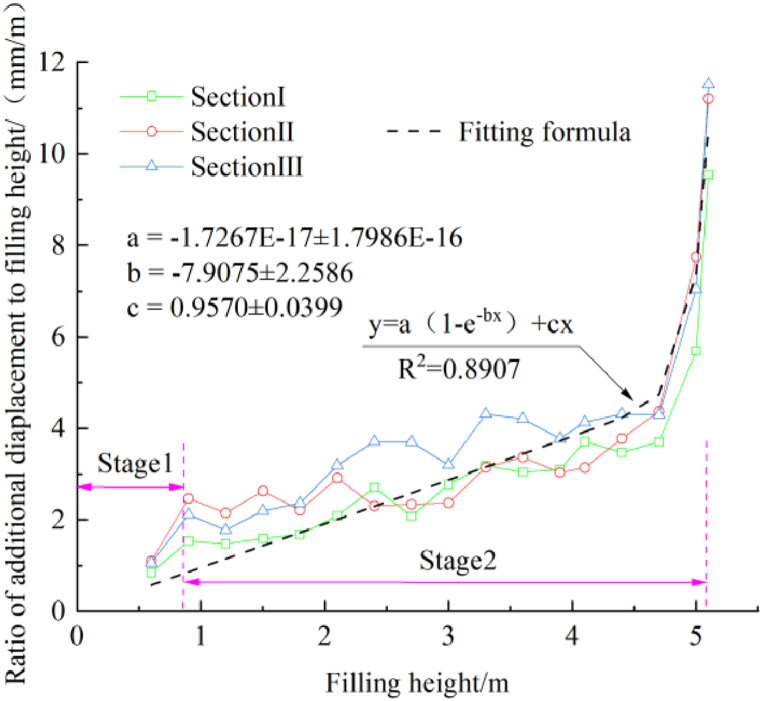
The relationship between the ratio of additional displacement to filling height with the filling height.

**Fig. 14:**
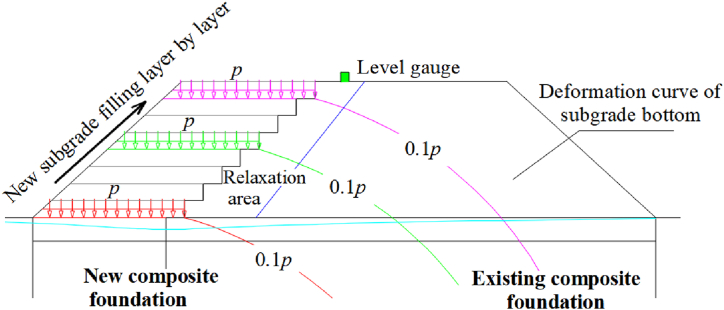
Schematic diagram of the additional stress contours of the existing subgrade under a uniform load for each layer.

Fig. 12 shows the displacement and filling height over time. The displacement in sections I, II, and III increases with the filling height. When the filling is completed, the displacement in sections I, II, and III is 13.716 mm, 15.948 mm, and 17.827 mm (≤5 cm). The results indicate that as the filling width of the new line increases, the influence on the existing line increases. The displacement has three stages: the lower filling stage, the intermittent stagnation stage, and the upper filling stage. In the lower filling stage, the rate of increase of the displacement increases with the filling height. In the intermittent stagnation stage, the rate of increase slows down due to soil viscosity. In the upper filling stage, the displacement increases rapidly. The relationship between the ratio of the additional displacement to filling height with the filling height is shown in Fig. 13. The ratio changes negligibly when the first layer has been completed. As the filling height increases, the ratio increases slowly. When the thickness of the filling is 0.5–0.8 m, the ratio increases sharply. Curve fitting provided the following equation: y = a(1-e^-bx^) + cx, R^2^ = 0.8907. As shown in Fig. 14, it is assumed that each layer has a uniformly distributed load. The influence on the existing subgrade increases layer by layer, and the influence magnitude on the existing subgrade level gauge increases. According to the literature [20,21] and our measurements, the settlement deformation of the existing subgrade is caused by two reasons. First, the load exerted on the widened subgrade filler is transmitted to the existing subgrade, resulting in additional stress and deformation. Second, the construction machinery causes disturbance and deformation during rolling. The growth rate of the additional deformation of the existing subgrade is higher at a higher filling height. Therefore, the deformation should be closely watched at the upper position during the construction of the new subgrade to ensure driving safety. Slight settlement deformation of the existing subgrade is observed in the intermittent stagnation period, which is related to the consolidation of the new subgrade and vehicles traveling on the upper side of the newly built subgrade.

## 4 Conclusions

Monitoring data was obtained during the construction of an adjacent railway subgrade in a loess area. The load exerted on the piles and the soil between the piles was analyzed, and the effect of the filling height on the earth pressure at the excavation steps was determined to investigate the influence of filling the new subgrade on the existing subgrade. The main conclusions were as follows:

(1) The stress at the pile top and between piles in the three sections increased with the subgrade filling height. Subsequently, it remained unchanged and decreased with the distance from the existing subgrade. However, the rate of increase differed in different stages. As the filling height increased, the soil load between the piles was transferred to the pile top.
(2) In the early stage of subgrade filling, the pile-soil stress ratio was close to 1 for all sections. The pile-soil stress ratio increased rapidly and the rate of increase slowed down when the filling height was 1.5–1.8 m (transition zone), and the changes differed for different sections. After the completion of subgrade filling, the pile-soil stress ratio was 3.336–3.926 in section I, 3.919–4.347 in section II, and 4.383–4.582 in section III.
(3) The vertical and lateral earth pressure at the steps in sections I to III increased with the filling height. The lateral earth pressure increased and decreased with the filling height in some locations, but basically increases with the increase of the filling height.
(4) The ratio of the vertical earth pressure to the upper filling thickness increased and decreased with the filling height. When the filling was completed, the ratio of the vertical earth pressure to the upper filling thickness was lower than γ_min_. The ratio of the lateral earth pressure to the thickness of the upper fill was greater than (γ*K_0_)_max_ at the beginning of the filling and then decreased with the filling height.
(5) The displacement of the level gauge in sections I, II, and III increased nonlinearly with the filling height. Curve fitting provided the equation y = a(1-e^-bx^) + cx to describe the relationship between the ratio of additional displacement to the height of the filling layer with the filling height. The influence on the existing subgrade increased with the filling width.

## 5 Limitations of the study

The study presents an experimental study on the load transfer mechanism and the influence of the adjacent railway subgrade in loess region. However, there are still some limitations to consider. Firstly, the existing railway is on operation during the construction of newly built railway, which results in the lack of influence analysis on various dimensions. Secondly, the study focuses on the certain work point in loess region, the variation is unknown when water level or geological condition changes.

## Data availability

All relevant data are within the paper.

## CRediT authorship contribution statement

**Wenhui Zhao:** Writing – original draft, Conceptualization. **Ke Zhang:** Formal analysis. **Jinjiang Xu:** Writing – review & editing. **Xueli Liu:** Supervision.

## Declaration of competing interest

The authors declare the following financial interests/personal relationships which may be considered as potential competing interests:Wenhui Zhao reports financial support was provided by 10.13039/501100001809National Natural Science Foundation of China. If there are other authors, they declare that they have no known competing financial interests or personal relationships that could have appeared to influence the work reported in this paper.
